# Rapid self-test of unprocessed viruses of SARS-CoV-2 and its variants in saliva by portable wireless graphene biosensor

**DOI:** 10.1073/pnas.2206521119

**Published:** 2022-06-28

**Authors:** Deependra Kumar Ban, Tyler Bodily, Abhijith G. Karkisaval, Yongliang Dong, Shreyam Natani, Anirudh Ramanathan, Armando Ramil, Sunil Srivastava, Prab Bandaru, Gennadi Glinsky, Ratnesh Lal

**Affiliations:** ^a^Department of Mechanical and Aerospace Engineering, University of California, San Diego, CA 92093;; ^b^Department of Bioengineering, University of California, San Diego, CA 92093;; ^c^Administration, Ampera Life, Inc., La Jolla, CA 92037;; ^d^Materials Science, University of California, San Diego, CA 92093;; ^e^Institute of Engineering in Medicine, University of California, San Diego, CA 92093

**Keywords:** aptamer, graphene, SARS-CoV-2, COVID-19, biosensor

## Abstract

We have developed a DNA aptamer-conjugated graphene field-effect transistor (GFET) biosensor platform to detect receptor-binding domain (RBD), nucleocapsid (N), and spike (S) proteins, as well as viral particles of original Severe acute respiratory syndrome coronavirus 2 (SARS-CoV-2) coronavirus and its variants in saliva samples. The GFET biosensor is a label-free, rapid (≤20 min), ultrasensitive handheld wireless readout device. The limit of detection (LoD) and the limit of quantitation (LoQ) of the sensor are 1.28 and 3.89 plaque-forming units (PFU)/mL for S protein and 1.45 and 4.39 PFU/mL for N protein, respectively. Cognate spike proteins of major variants of concern (N501Y, D614G, Y453F, Omicron-B1.1.529) showed sensor response ≥40 mV from the control (aptamer alone) for fM to nM concentration range. The sensor response was significantly lower for viral particles and cognate proteins of Middle East Respiratory Syndrome (MERS) compared to SARS-CoV-2, indicating the specificity of the diagnostic platform for SARS-CoV-2 vs. MERS viral proteins. During the early phase of the pandemic, the GFET sensor response agreed with RT-PCR data for oral human samples, as determined by the negative percent agreement (NPA) and positive percent agreement (PPA). During the recent Delta/Omicron wave, the GFET sensor also reliably distinguished positive and negative clinical saliva samples. Although the sensitivity is lower during the later pandemic phase, the GFET-defined positivity rate is in statistically close alignment with the epidemiological population-scale data. Thus, the aptamer-based GFET biosensor has a high level of precision in clinically and epidemiologically significant SARS-CoV-2 variant detection. This universal pathogen-sensing platform is amenable for a broad range of public health applications and real-time environmental monitoring.

## Significance

“Am I positive or negative?” Everyone wants to know the answer with speed and accuracy. Rapid and accurate at-home testing is the best defense against the COVID-19 pandemic and ensuing endemics. Current rapid tests are often imprecise, test for denatured and processed viral components, and lack specificity for new variants. We developed a simple at-home test using saliva swabs that answers “positive or negative” in minutes and transmits results to stakeholders. The test uses a DNA aptamer-derivatized graphene field-effect transistor (GFET) to detect unprocessed intact SARS-CoV-2 and its variants at levels as low as 7 to 10 viruses. This method is tunable and adaptable for early-stage detection of emerging viral infections as well as many diseases with accessible biofluids.

The triad of testing, isolation, and vaccination is essential for minimizing the impact of the COVID-19 pandemic on an individual as well as on a global scale (1–3). A standard high-precision PCR test for COVID-19 viral infection requires several steps of biochemical processing (4) and is time consuming (average reporting time is 24 h or longer) and resource intensive. It causes significant delays in management and containment of viral spread and treatment (5, 6). Recently developed CRISPR-Cas–based techniques, while highly sensitive (zM to aM), rely on complex amplification methods such as Recombinase polymerase amplification (RPA), Reverse transcription loop-mediated isothermal amplification (RT-LAMP) (7, 8), or multiple probes (e.g., up to 10 CRISPR-RNA (crRNA) and a fluorescence probe) for detection (9).

A rapid, accurate, and simple detection and diagnostics strategy, ideally incorporating a mobile handheld point-of-care (POC) platform with instant wireless data reporting, has emerged as the most desirable testing concept to control the spread of viral infection (10). Rapid testing using antigens and antibody-based assays often suffers from false results due to the assays’ quality and denaturation of the antibody outside laboratory settings, as well as nonspecific interactions with viral constituent molecules (11–15). More accurate and sensitive aptamer-based detection methodologies (16–19) usually involve biochemical assays and surface plasmon resonance (SPR) spectroscopy, a colorimetric lateral-flow test (17). The biochemical assays (19) are limited by lower sensitivity, sample processing, and a multistep detection process. Biochemical sensors, fabricated by attaching specific reactive molecules, need prior information about interfering agents in the samples (19). An aptamer-based graphene field-effect transistor (GFET) system has been used to detect Severe acute respiratory syndrome coronavirus 2 (SARS-CoV-2) RNA (20). However, no direct detection of intact SARS-CoV-2 virus particles is available, although the whole viral particles are the most infective agent.

**Fig. 1.**
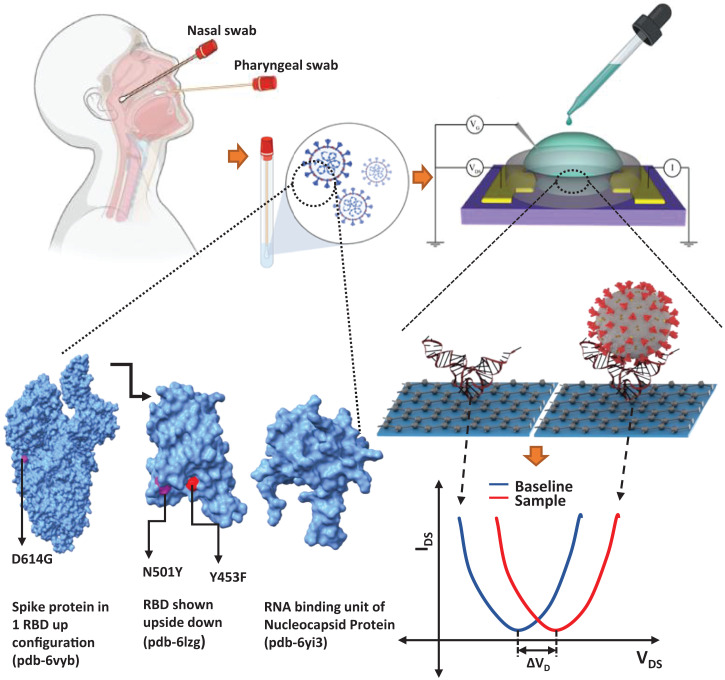
Schematic summarizes the Aptamer-GFET sensor diagnostic system for ultrasensitive detection of SARS-CoV-2 using patient samples. (*Upper Left*) Sample collection scheme. (*Upper Right* and *Lower Right*) Schematic of GFET sensor workflow. Plot shows a typical GFET sensor test output with baseline and sample curves. The graphic below shows the sensor layout and electrical circuit. The diagram below shows a cartoon representation of binding interaction between the 3D folded aptamer and RBD protein as an example (note that the aptamer structure shown is purely for representation/visualization purposes only and does not represent the actual 3D structure acquired by the aptamer, not to scale; refer to *SI Appendix*, Fig. S1 for 2D equilibrium secondary structures of the aptamers). (*Lower Left* to *Center*) Protein Data Bank structures of spike (6VYB), RBD proteins (6LZG), and RNA-binding domain of nucleocapsid protein (6YI3) shown with mutated residues marked using University of California, San Francisco, ChimeraX (50).

In the present work, we describe design, manufacturing, and analytical performance of GFET-based biosensors for viral detection (21–24), with advancement over our earlier-reported nucleic acid conjugation and single-nucleotide resolution detection capability, to detect SARS-CoV-2 antigen using DNA aptamers. The method is based on the highly specific interactions of diagnostic targets of interest, e.g., unprocessed SARS-CoV-2 viruses present in a saliva sample and DNA aptamer probe-derivatized GFETs. The interaction of aptamer (probe) with virus (analyte) modulates the interfacial electric field arising from the captured aptamer–virus complexes. The sensitivity of testing relies upon the sensitivity of the graphene surface and its surface defects, including crumpling (25–27). The related changes in the current (28), voltage, and capacitance (29) provide a self-consistent record of the detected moiety (Fig. 1). However, detection of analytes in a biological buffer is limited by Debye screening length (∼ <1 nm) (30–32). Aptamers containing a small number of nucleotides after three-dimensional (3D) folding reduces the distance between the graphene and the sensing part of the probe and thus being in the overlapping Debye screening length increases the sensitivity. The smaller size, higher affinity, stability compared to antibodies (more than 100 kDa, size >10 nm), and easy quality control after the systematic evolution of ligands by exponential enrichment (SELEX) process (33) make the aptamer an optimal candidate for developing a SARS-CoV-2 biosensor.

We report the detection of SARS-CoV-2 viral particles and its antigens of major variants of concern (N501Y, D614G, Y453F, and Omicron) (Fig. 1). SARS-CoV-2 coronavirus spike (S)- and nucleocapsid (N)-protein–specific aptamers (K_d_ ∼ 5.8 nM) (16) and (K_d_ ∼ 0.5 nM) (17), respectively, were able to reliably detect SARS-CoV-2 antigens and viral particles. Both aptamers were modified for GFET derivatization at the 3′ end to functionalize the graphene surface. We were able to use these aptamer-derivatized GFET biosensors to identify S-protein receptor-binding domain (RBD) and N protein using cognate proteins, viral particles, and patients’ oral swab samples collected in the early phase of the pandemic as well as saliva samples collected during the recent Delta/Omicron wave. The pathogen detection principles and the technology outlined here will serve as readily available POC diagnostics. The mass-scale deployment of the reported POC sensor would help considerably in controlling current and preventing future viral outbreaks.

## Results and Discussion

### GFET Characterization.

**Fig. 2.**
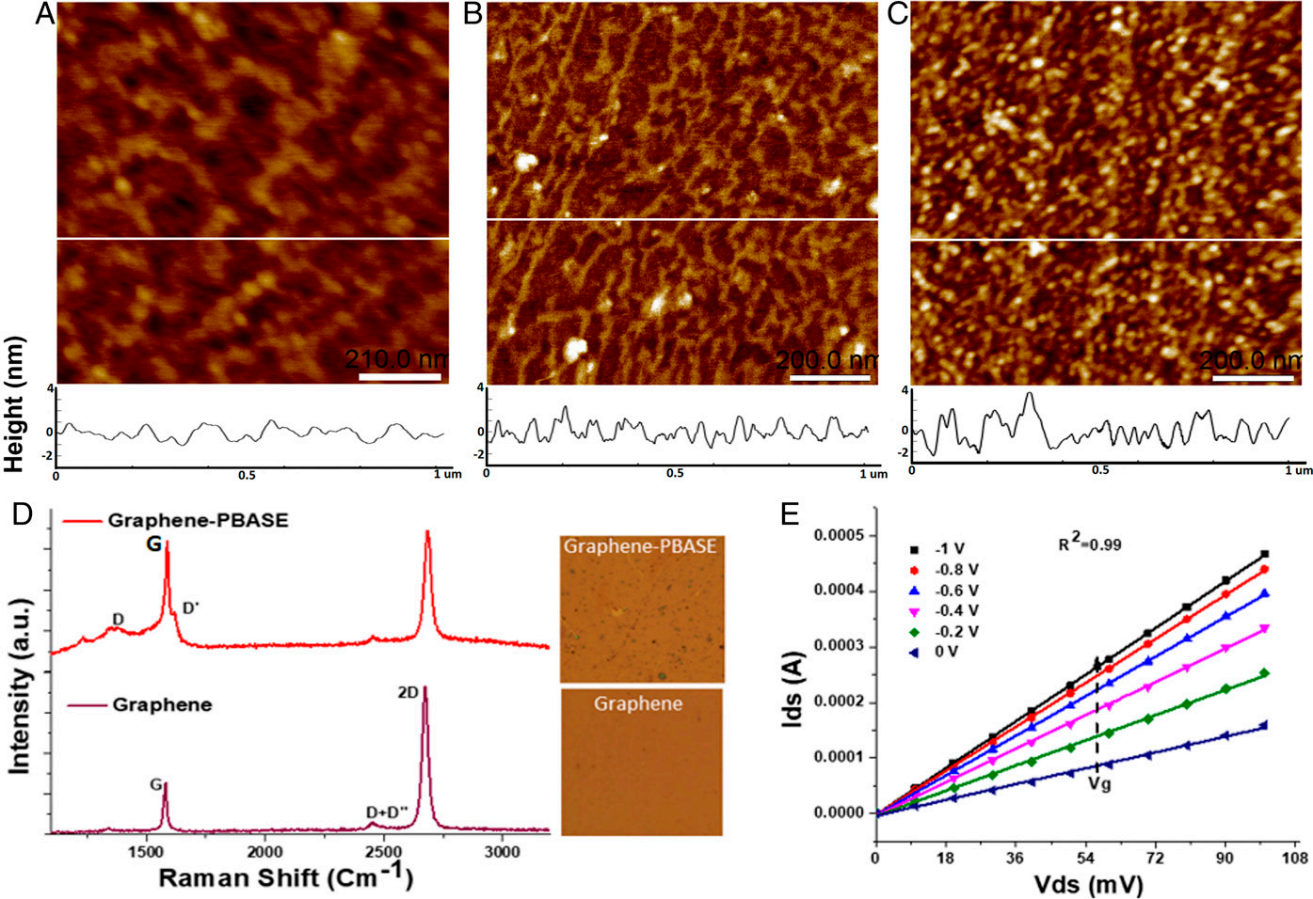
GFET characterization—atomic force microscopy height mode images of GFET surface after different steps: (*A*) bare graphene surface, (*B*) after 5 mM PBASE addition, and (*C*) after 1 μM aptamer functionalization. (Scale bars, 200 nm; z scale in all images, 0 to 7.5 nm.) The white horizontal line in each image represents a section through the image whose height profiles (in nanometers) are included below each respective image. (*D*) Raman spectroscopy of pristine graphene and PBASE-functionalized GFET (*Inset* shows light microscope image of scanned area). (*E*) Transfer characteristics (*I_DS_* vs*. V_DS_*) of GFET derivatized using PBASE (5 mM) were analyzed by sweeping *V_GS_* from 0 to −1 V at 0.2V steps and variable drain source voltage (*V_DS_*) of 0 to 100 mV with incremental steps of 10 mV.

The quality of the graphene surface has a major impact on the GFET performance (25). To characterize the quality of the graphene surface in the GFET sensor and the effect of subsequent derivatization steps, we imaged the surface by atomic force microscopy (AFM) in contact mode. The wet transfer process leads to graphene being deposited as interconnected grains with defined grain boundaries that are visible at lower magnifications (*SI Appendix*, Fig. S5). High-magnification images of pristine graphene surface (Fig. 2*A*) and after 1-pyrenebutyric acid *N*-hydroxysuccinimide ester (PBASE) derivatization (Fig. 2*B*) show that the graphene surface is flat at the nanoscale with root mean square (RMS) roughness of 0.7 ± 0.2 nm and after PBASE derivatization the surface roughness changes to 1.4 ± 0.3 nm. All subsequent derivatization/attachment steps increased surface roughness. Corresponding Raman spectroscopic analysis of the GFET surface is shown in Fig. 2*D*. Pristine graphene shows the expected G and 2D peaks with a peak intensity ratio greater than 1:2, indicating a single-layer characteristic, for the graphene. After derivatization with PBASE, the appearance of D and D′ peaks in the spectra can be attributed to pyrene group binding and related enhanced sp^2^ bonding (27, 34).

We characterized the GFET by measuring the *I_DS_* at sequential *V_DS_* values while maintaining *V_GS_* at a constant value (Fig. 2*D*). A linear variation of the *I_DS_ – V_DS_* (output curve) was observed, which indicates Ohmic characteristics at all the tested *V_GS_* values. GFET chips were sourced from three different providers, using the same design layout and overall manufacturing process; however, a slight variability in performance can be expected. Hence, chip-to-chip variability in performance is expected without compromising the detection/sensing capability.

To optimize the performance of each GFET chip and to minimize the interchip variabilities, reasonable quality control measures were adopted. As wet transfer of graphene onto the wafer can introduce variability, batchwise quality checks were done by examining the Raman spectra of graphene to ensure optimal transfer of graphene and confirming its single-layer presence. However, this still does not ensure total uniformity among all the chips at a microscopic level and after the metal contacts and wire bonding, an additional variability can be expected due to contact resistances. To ensure consistent performance, we considered only chips that had a final resistance of ≤8 kΩ. This resistance threshold is conservative and based upon the current measurement data from more than 500 manufactured GFET chips and using both our portable reader (called pathogen investigation via Ohmic technologies [PIVOT]) and Keithley 2400/2602B source meters. With these constraints in mind, we emphasize that the absolute value of Dirac voltage shift calculated from one chip under certain experimental conditions should not be directly compared to another chip. However, each GFET chip is a self-consistent record of the test: all Dirac voltage shifts reported in the data are from each individual chip’s baseline reading and hence the determination of analyte’s presence is solely decided by considering the difference from the baseline response of each chip. Each chip can give a different amount of shift for the same aptamer and same target analyte at the same concentration (within a defined range to exclude outliers). The binding “discriminatory criterion” is the voltage difference solely from its previous step on the same GFET chip.

### Detection of SARS-CoV-2 and Its Mutant Antigens.

**Fig. 3.**
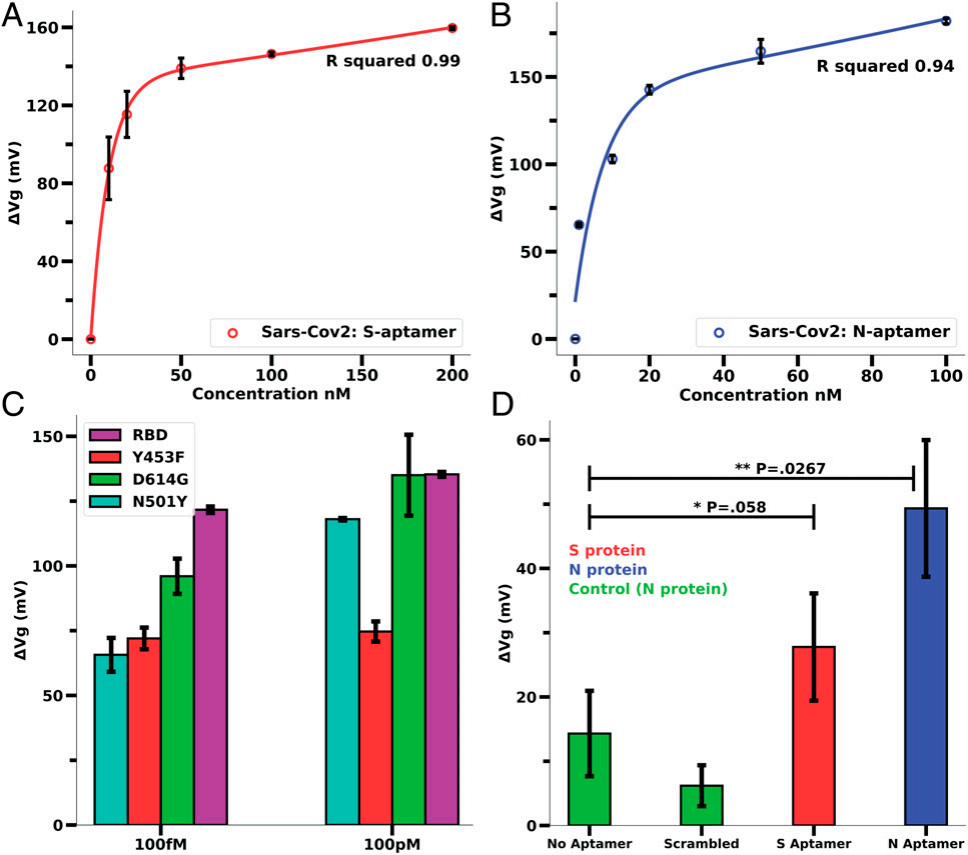
Concentration dependence and cognate protein response of Aptamer-S and Aptamer-N–derivatized GFET sensor. (*A*) RBD concentration (0 to 200 nM)-dependent sensor response on Aptamer-S–derivatized GFET. (*B*) N-protein concentration (0 to 100 nM)-dependent sensor response on Aptamer-S–derivatized GFET. (Refer to *SI Appendix*, Fig. S6 for normalized *I_DS_ vs. V_GS_* curves.) (*C*) Aptamer-S–derivatized GFET sensors were able to detect B.1.1.7 variant (N501Y), mink-related mutation (Y453F), and mutation at S2 domain (D614G) of SARS-Cov-2. Error bars of *A–C* were calculated from data from three different chips. (Refer to *SI Appendix*, Fig. S7 for normalized I_DS_ vs. V_GS_ curves.) (*D*) Response of the N- and S-aptamer–derivatized GFET sensors for S and N proteins (cognate, 100 nM concentration) of the Omicron variant (B1.1.529). *P* values are generated from a two-tailed Student’s *t* test: **P < 0.05, *P < 0.1. The error bars for *D* were calculated using data from two chips for each bar.

Due to a wide range of viral load in a patient sample (10^4^ to 10^7^ copies per milliliter and 3.5 fM to 58.9 nM of antigen level) (35–37), it would be highly important to analyze the concentration-dependent sensitivity as well as the saturation of the GFET sensor response for reliable detection. The analyses of Aptamer-S– and -N–derivatized GFET sensor responses for different concentrations of RBD and N protein of SARS-CoV-2 indicate a concentration-dependent exponential shift in the Δ*V_D_* (or sensor response). The sensor reached saturation at 200 nM of RBD and 100 nM of N protein. (Fig. 3 *A* and *B*). Significantly, both S and N proteins when tested at a wide concentration range produced a ≥40-mV Dirac voltage shift compared to the baseline reading. Moreover, the Dirac potential saturated for higher concentrations of N and S proteins and after saturation, the excess antigen level did not reduce the sensor response.

Current antigen tests approved by the Food and Drug Administration (FDA) under emergency use authorization (EUA) (*SI Appendix*, Table S2) did not indicate the ability to detect new variant(s) that may escape immunity generated by available vaccines or past infection (38). There has also been considerable concern about false negatives of the recommended tests (39). The evolution of new mutations of SARS-CoV-2 (B.1.1.7 variant [N501Y], mink-related mutation [Y453F], mutation at S2 domain [D614G], B1.1.529 [Omicron]) and other emerging variants is of major global concern (40). Considering the importance of such issues, we deployed the GFET sensor on different SARS-CoV-2 mutant antigen proteins. Results indicate that Aptamer-S showed more than 40 mV of sensor response in the 100-fM to 100-nM concentration range of respective proteins (Fig. 3*C*). Although there is variant-specific variability in sensor response observed, however, sensor response was always above the threshold value for positive sample (i.e., >40 mV) (Fig. 3*C* and *SI Appendix*, Fig. S3). After the inclusion of the ionic drift incubation step there was an overall minor decrease in sensor response (∼15%) but it was still consistent across control and sample measurements. Based on the concentration-dependent analysis of cognate RBD and N protein on respective aptamer-derivatized GFET sensors with the SARS-CoV-2 variant, it strongly supports the conclusion that our GFET sensor can detect relevant viral antigen at fM to nM concentration levels. We anticipate the enhanced sensitivity, as well as the concomitant specificity, is due to nonoverlapping binding sites of aptamer-S at spike protein amino acids T500, N437, and Q506 (16) with new mutations.

Omicron N and S proteins were also tested against the N and S aptamers, respectively. The results (Fig. 3*D*) indicate a reduction in signal from the RBD protein of the wild-type virus, as Student’s t-test indicates a *P* value of 0.058 between S and the control shift and 0.0267 between N and the control. This is an expected result as the RBD of the Omicron variant has about 15 mutations and overall, the spike protein has more than 30 mutations that lead to decreased binding with the aptamer (41). It should be noted that the signal is still sufficiently distinguishable between responses with and without addition of antigen proteins but an overall reduction in signal is noticeable.

### Analyzing the Sensor Specificity.

**Fig. 4.**
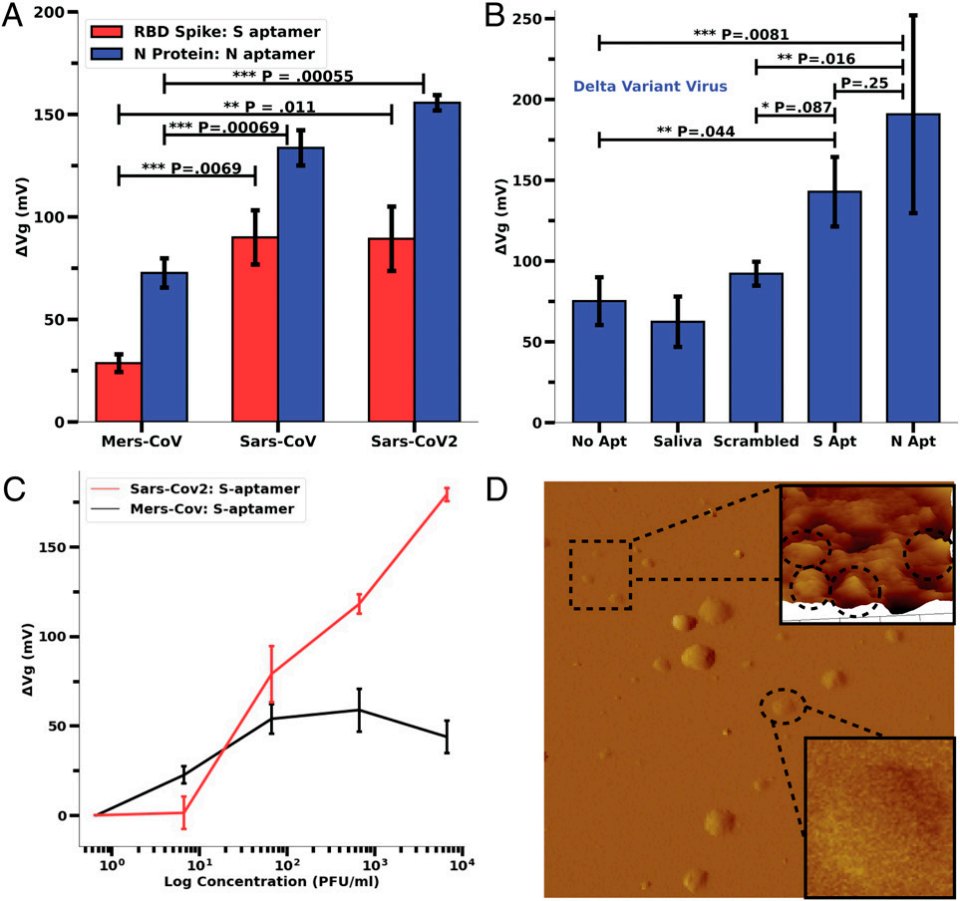
Specificity analysis and GFET response to inactivated viruses. (*A*) A total of 100 pM of RBD protein and N protein of MERS, SARS, and SARS-CoV-2 were used on Aptamer-S– and -N–derived GFET, respectively, to analyze the specificity of both the aptamers. It was indicated that both the aptamers were able to differentiate RBD and N protein of MERS but were unable to differentiate between SARS-CoV and SARS-CoV-2. Each error bar was calculated from data from three different chips. (Refer to *SI Appendix*, Fig. S8 for normalized *I_DS_* vs. *V_GS_* curves.) (*B*) Response of Aptamer (Aptamer-S, and -N)–derivatized GFET with heat-inactivated SARS-CoV-2 Delta variant virus (2 × 10^8^ copies per milliliter ORF1a) in saliva diluted in PBS buffer (10,000× dilution). *P* values are generated from a two-tailed Student’s *t* test: ***P < 0.01, **P < 0.05, *P < 0.1; ns, *P* > 0.1 or not significant. Error bars were calculated using several different chip results; no aptamer, saliva, and scrambled aptamer control results each had two chips. The error bars for the S aptamer and N aptamer are composed of three and five chip results, respectively. (*C*) Linear-log plot of concentration dependence and relative response of MERS-CoV and SARS-CoV-2 inactivated viruses shown as absolute Dirac voltage shift. Error bars have been calculated from three different chips. (*D*) AFM height image of inactivated delta variant sample on poly-l-lysine–coated mica. (Scan size, 10 × 10 μm, z scale = 100 nm.) *Inset* shows a higher-magnification area with few particles ranging in lateral size from 100 to 150 nm. In the larger area scan, clusters/aggregates of viral particles are found, with some individual particles in the 100- to 150-nm lateral size range.

To test the specificity of our sensor with aptamer-S and -N, we have used closely correlated cognate antigen of MERS-CoV, SARS-CoV, and SARS-CoV-2 as well as inactivated Middle Eastern Respiratory Syndrome corona virus (MERS-CoV) and SARS-CoV-2 viruses. The results clearly show that aptamer-S and -N significantly differentiate the MERS-CoV and the SARS virus proteins (>40 mV increment of sensor response, *P* < 0.05) (Fig. 4*A*). However, the GFET was unable to significantly differentiate between SARS-CoV and CoV-2 proteins (both N and S, *P* > 0.1). Also, the maximum sensor response from Aptamer-N (Fig. 4*A*, red bars, 50%) is higher with all tested protein samples compared to Aptamer-S (Fig. 4*A*, blue bars, 35%) and may be ascribed to higher affinity of aptamer (K_d_ ∼ 0.5 nM compared to K_d_ ∼ 5.8 nM for Aptamer-S).

To verify the specificity of aptamers and sensitivity to detect intact viral particles, we analyzed the sensor response by applying a 10,000× dilution of heat-inactivated delta variant of SARS-CoV-2 (Fig. 4*B*). Results indicate that both S- and N-aptamer–derivatized chips show a distinguishable response compared to a scrambled variant of the aptamer with N-aptamer–derivatized chips showing more change in Δ*V_D_* compared to S aptamer (*P* = 0.016 N aptamer, *P* = 0.085 S aptamer). The overall response is slightly reduced, which can be attributed in part to the fact that the delta variant spike protein contains a larger number of mutations than the N protein. The overall sensor response for the N aptamer and the S aptamer has a distinguishable peak from the control experiments. To further analyze the specificity of the Aptamer-S– and -N–derivatized chip in simulated biological conditions, we prepared different equivalent dilutions of inactivated SARS-CoV-2 and MERS-CoV in 10× vol/vol saliva in 1× phosphate-buffered saline (PBS) buffer. The reason for higher sensor response with SARS-CoV and SARS-CoV-2 viruses might be the high affinity of aptamer-S at RBD amino acid positions 500, 437, and 506 containing threonine (T), asparagine (N), and glutamine (Q) while low sensor response with MERS might be due to presence of different amino acids (alanine [A], lysine [K], and alanine [A]) at the same position of RBD (16, 42).

This finding clearly signifies that both (Aptamer-S and -N) functionalized sensors are specific for SARS-CoV-2 proteins of the virus. However, the GFET sensor with aptamer-S usually showed higher sensor response compared to aptamer-N in simulated biological samples for the original SARS-CoV-2. Most likely this is reflective of the likelihood of the surface S protein being more easily accessible compared to N protein that is encapsulated within the viral envelope (43, 44).

### Limit of Detection and Limit of Quantification.

**Fig. 5.**
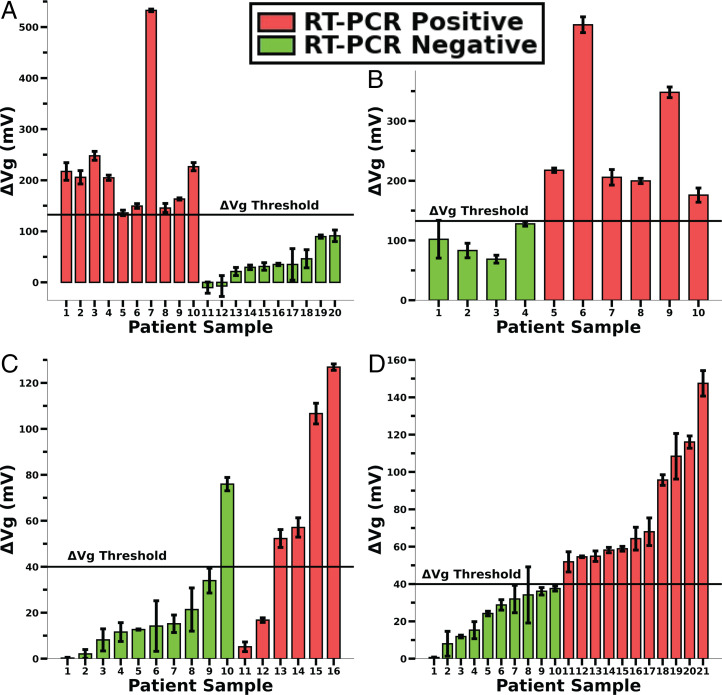
Patient sample analysis—wild-type and Omicron variants. (*A*) Dirac voltage shifts from 20 patient oral swabs. Ten confirmed RT-PCR–positive, Ct ≤ 35 (red bars), and 10 confirmed RT-PCR–negative patients (green bars) were collected during the first wave of COVID-19 (wild type/Chicago, IL; March 2021). (*B*) Blind sample test (as described in *Materials and Methods*, *On-Field Clinical Sample Testing*) using aptamer-S–derivatized GFET. (*C*) Dirac voltage shifts from 16 patient saliva samples, 10 confirmed RT-PCR–negative (green bars) and 6 confirmed RT-PCR–positive patients, avg Ct < 29.14 (red bars), were collected during the Delta/Omicron variant wave (Las Vegas, January 2022). (*D*) Prediction of remaining collected patients’ saliva samples (Omicron) using a threshold voltage value of 40 mV (green bars, negative; red bars, positive). *A* and *B* have error bars from five different sweeps on the same GFET chip whereas *C* and *D* have error bars calculated from three different *I_DS_ vs. V_GS_* sweeps on a single chip.

To analyze the limit of detection (LoD) and limit of quantification (LoQ) of our aptamer-based sensor, we have performed concentration-dependent sensor response (Fig. 4*D*) analysis, with inactive viruses in simulated biological conditions. Triplicate data per test case were fitted using linear fit and the LoD and LoQ were calculated following FDA statistical data analysis guidelines as described in *Materials and Methods*. The results indicated a LoD with aptamer-S of 1.28 plaque-forming units (PFU)/mL (*R*^2^ = 0.98) and that with aptamer-N of 1.45 PFU/mL (*R*^2^ = 0.99) with inactive virus in 10% vol/vol saliva and 1× PBS buffer at room temperature (Fig. 5 *A* and *B*). The estimated LoQ was 3.89 and 4.39 PFU/mL for aptamer-S and -N, respectively. Our test showed higher sensitivity (lower LoD) compared to the FDA-approved antigen test (*SI Appendix*, Table S2). These results clearly indicate the sensitivity of the aptamer-based GFET sensor to detect SARS-CoV-2 in biological fluids and provide motivation for investigation with real patient samples.

### Clinical Sample Analysis.

Based on the simulated biological sample analysis, it was observed that aptamer-S–derivatized GFET showed high sensor response while aptamer-N response was smaller (Fig. 4*D*). Moreover, since the original SARS-CoV-2 virus has only a few defined mutations in S protein, we used aptamer-S–derivatized GFET for patient sample analysis (during the initial testing in March 2021) as described in *Materials and Methods* for clinical data analysis during the first phase of clinical sample analysis. To validate the GFET sensor with related samples, a single blind experiment was performed with 10 each negative and positive samples and confirmed independently through RT-PCR (Fig. 5 *A* and *C*). Moreover, 10 double-blind samples were tested to confirm the test. Based on the results, we first used RT-PCR negative samples to predict with a CI of 99.7% +3σ of sensor response, which yields 132.6 mV of the *V_D_* shift for the samples. A threshold value of 132.6 mV was set with a *V_D_* shift above this threshold considered as positive confirmation of SARS-CoV-2 infection (Fig. 5 *A* and *C* and *SI Appendix*, Table S3). Further, we performed the negative percent agreement (NPA) and positive percent agreement (PPA) analyses as described in *Materials and Methods*. A retrospective comparison of FDA-approved Promega RT-PCR tests for the SARS-CoV-2 test showed 100% NPA and 100% PPA as related to our GFET-based derivatized sensor. It is expected that including more samples and emergence of new variants will decrease the NPA and PPA values. We also expect wide variability in virus load and composition of collected patient samples, variation in mutation-mediated charge distribution on the viral protein surface, and aptamer–virus particle-binding affinity. This was reflected during the recent set of testing during the Delta/Omicron wave that shows higher variability in sensor response (Fig. 5 *C* and *D*).

For clinical samples tested during the prevalence of Delta/Omicron variants, the variability in sample states (viral load, baseline saliva constituency being variable among individuals) caused a wider variability in the GFET sensor response. A minority of GFET sensors had high Dirac voltage shifts, although their corresponding RT-PCR results confirmed them to be negative samples (Fig. 5*C*, P10). Taking an average value of the Dirac shifts for threshold calculation can easily skew the results as average values are sensitive to outlier data. To account for this variable, we calculated a more conservative statistical test, the interquartile range (IQR), and used 1.5*IQR as the upper limit over the 75% quartile value of the dataset. If a Dirac shift value crossed this upper threshold, they were considered outliers. Calculating IQR revealed that sample 10 (P10) was an outlier compared to samples 1 to 9 and hence sample 10 was excluded from threshold calculation for Delta/Omicron variants in Fig. 5*C*. After P10’s exclusion, the 3σ threshold for positive vs. negative discrimination was found to be 35.1 mV. We used 40 mV as a conservative threshold value for future analyses of predictive clinical sample results.

Recent clinical trials using the PIVOT reader and the GFET during the Delta/Omicron wave showed >40 mV of sensor response for 3 of 5 RT-PCR–positive patient samples (Fig. 5*B*) and 1 of the 10 RT-PCR samples was negative. In the first round of clinical tests (during the original SARS-CoV-2 pandemic phase), a threshold of ∼130 mV was a sufficient Dirac voltage shift to distinguish the negative and positive samples. The Dirac voltage shift obtained from recent testing (Delta/Omicron pandemic phase) indicates only a maximum of 120 mV. When a >40 mV Dirac voltage shift was used as the threshold to distinguish positive from negative infection, the calculated PPA is 66% and NPA is 90% for samples tested during the second phase. The reduction in sensitivity could be due to the high number of mutations acquired by the virus Delta/Omicron variants (44). Considering the updated threshold voltage, we predicted the positivity rate of the remaining patient samples (RT-PCR data were not available for these samples) and the results are displayed in Fig. 5*D*. At a 40 mV cutoff, the GFET sensor predicted 10 negative and 11 positive samples. Of note, the GFET sensor-predicted positivity rate of 52% appears to correspond to contemporary epidemiological findings of the infection prevalence in the population (Centers for Disease Control [CDC] Daily Report).

Using analytical optimization protocols for data processing, exclusion, and discrimination threshold considerations, we conclude that GFET’s PPA and NPA appear to be reduced to 67% and 90%, respectively, for recently collected patients’ saliva samples during the Delta/Omicron wave. These findings may also reflect the fact that the aptamers used to test these potential Delta/Omicron viruses were selected against the proteins of the wild-type virus and, therefore, might have somewhat reduced affinity toward new variants harboring multiple mutations. Future studies with newly designed aptamers to be specific to each coronavirus variant may help to resolve these uncertainties. Nevertheless, it is very encouraging that aptamers selected against the proteins of the wild-type virus circulating in the human population 2 years ago retained high detection capacity toward mutant viral proteins and mutant viruses circulating in the human population at the present time.

To our knowledge, the aptamer-based GFET devices presented in this contribution demonstrate superior analytical characteristics compared to currently available on-the-market FDA-approved COVID-19 diagnostics. These conclusions are valid in terms of the simplicity of sample preparation and processing requirements, which eliminates extraction and/or enzymatic digestion steps, facilitating the simple, end-user–friendly data acquisition protocol. Additional state-of-the-art features include comprehensive user-independent real-time wireless data-reporting systems, including not only the test results but also smart phone-enabled real-time device location identification and contact-tracing capabilities. The analytical characteristics of our GFET devices, including the sensitivity and specificity features, appear to exceed the performance characteristics of FDA-approved COVID-19 diagnostics currently available on the market (*SI Appendix*, Table S2).

## Conclusion

Based on the cognate antigen analysis, both the S and N aptamers can detect fM to nM concentrations of RBD and nucleocapsid proteins of SARS-CoV-2. Dirac voltage shift analyses vs. the control (aptamer alone) from GFET sensors indicate the significant detection capabilities of RBD and spike proteins of wild-type SARS-CoV-2 and mutant variants of concern. Specificity analysis indicates that both aptamers were able to differentiate the MERS-CoV protein from SARS-CoV-2 but could not distinguish between SARS-CoV and SARS-CoV-2. Laboratory tests with inactivated wild-type and mutant viruses confirm that the GFETs are clearly capable of differentiating viral samples in the buffer and simulated clinical environments of human saliva samples. As a further confirmation of the on-field efficacy and potential POC application utility of these sensors, clinical sample tests on GFET sensors validated by RT-PCR results indicate a clear distinction between positive and negative samples at respective threshold voltages.

In summary, our GFET sensor response analysis with SARS-CoV-2 cognate proteins, further extended to both inactivated wild-type and mutant viruses in simulated clinically relevant conditions and patients’ samples, was successful in detecting SARS-CoV-2 coronavirus and its molecular moieties. The GFET sensor-defined diagnosis of the SARS-CoV-2 infection is highly sensitive and rapid (<20 min) with high specificity. Based on a comparative analysis of FDA-EUA–approved COVID-19 diagnostic tests (*SI Appendix*, Table S2), the reported analytical endpoints of the proposed GFET sensor are comparable with best-performing tests, including PCR-based diagnostics. Our GFET sensor and related handheld devices can be implemented in practice as a readily available platform for POC diagnostics, and the aptamer-based methodologies may be adapted for other disease diagnostics in the future by functionalization with other aptamers optimized for targets of interest.

## Materials and Methods

### Materials.

SARS-CoV-2, MERS, and SARS-CoV cognate nucleocapsid (hereafter called N protein) and the receptor binding domain of the spike protein (hereafter called RBD protein) were purchased from Sino Biological. HPLC grade 3′ amino functionalized aptamers for N protein (Aptamer-N) (17) and spike RBD (Aptamer-S) (16) probes were designed to functionalize the graphene surface of the GFET and purchased from Integrated DNA Technology (IDT). Molecular biology grade 1× PBS (Gibco no. 10010031), MgCl_2_ (Invitrogen no. AM9530G), 1× normal saline (McKesson no. 37-6281), and ultrapure water (Invitrogen no. 10977015) were used throughout the study. Analytical-grade PBASE (Anaspec Inc. no. AS-81238) and ethanolamine (Alfa Aesar) were used without further processing. Inactivated SARS-Cov-2 USWA1/2020 isolates (9.55 × 10^6^ TCID_50_/mL) were purchased from Zeptometrix Co. along with the inactivated delta variant (B.1.617.2) and used after dilution in 1× PBS/0.9% saline in the recommended BSL-II laboratory facility. Omicron nucleocapsid and spike proteins were purchased from Acro Biosystems (N, no. NUN-C52Ht, and RBD, no. S1N-C52Ha).

### Graphene Field-Effect Transistor Fabrication and Characterization.

Graphene was synthesized on 25-µm-thick copper foil (MTI Corp.) by low-pressure chemical vapor deposition (LPCVD). The LPCVD-grown graphene on copper was spin coated at 3,000 rpm for 45 s by 120-K molecular weight polymethyl methacrylate (PMMA) for a PMMA-assisted wet transfer process. Oxygen plasma etching was applied to remove the graphene on the backside of the copper foil. Ammonium persulfate (0.1 M) solution was used to etch copper foil and subsequently rinsed with deionised (DI) water. The Poly(methyl methacrylate) (PMMA)/graphene stack was transferred on a patterned SiO_2_/Si substrate with channel length of 500 µm with ∼100-nm-thick Au/Cr electrodes prepared by sputter deposition. The PMMA was dissolved using acetone for 1 h followed by isopropyl alcohol (IPA) rinse and was nitrogen blow-dried. To define the graphene channel, photolithographic micropatterning techniques with (Poly-Methyl Glutarimide) PMGI photoresist were applied to protect the graphene channel, and extra graphene was removed by oxygen plasma etching. The GFET assembly was further annealed at 200 °C for 2 h under a forming gas atmosphere to anneal impurities. The fabricated chip was glued to a printed circuit board (PCB) board/chip carrier and the gate, source, and drain terminals were wire bonded to the contact pads. The source and drain Au/Cr electric pads and the wire bonds were protected from direct contact with the electrolyte solution using silicone paste and a well (3 to 5 mm internal diameter) made of epoxy or silicone tubing was glued onto the chip, which acted as a reservoir during derivatization and sample incubation (*SI Appendix*, Fig. S2).

The surface quality of the graphene incorporates several elements related to 1) the graphene continuity, with the possibility of nanoscale pores; 2) the roughness of the graphene surface, which may present a concave (convex) surface to the analyte with increased (decreased) sensitivity; 3) the amount of wrinkles, which may cause localized electron puddle formation; 4) characteristics of the edges of the deployed graphene, which may induce a localized metallic/insulating character; and 5) the extent of impurities or defects present on the surface, along with their charge (positive/negative/neutral).

Roughness, as indicated earlier, or wrinkling of the graphene surface may either decrease or increase the charge sensitivity. For instance, Debye length screening, related to the GFET response, would be stronger (weaker) near surfaces with convex (concave) curvature. A weaker screening could be interpreted in terms of a smaller capacitance density and induce greater sensitivity. Such aspects have been further delineated in the literature (45).

The surface roughness measures indicated in the text, e.g., 0.7 ± 0.2 nm, for the bare graphene surface, and 1.4 ± 0.3 nm, after PBASE derivatization, were obtained through AFM scans on representative areas of the graphene and derivatized surfaces, respectively.

As a rough quality control metric for GFET fabrication, source-drain resistance (*R_SD_*) was measured on the chips and those above 8 kΩ were excluded from the data collection process. The reader was tuned for optimal current measurement when *R_SD_* is less than 8 kΩ. The GFET sensors used in this study were from three different sources (University of California, San Diego and two other external manufacturers) using the same design and integrated with the fluid sample well. The integrated GFET chips were not optimized. The success rates for these devices to obtain a complete set of data for a specific analyte–probe interaction (e.g., S aptamer and S protein) were ∼25 to 30%; the defective devices were discarded and data from these devices are not included in our analysis. All data presented in this article use each chip compared with its own internal control (i.e., data with aptamer alone as a baseline control and the data after adding an analyte [e.g., viral proteins, inactivated virus saliva sample]). Incorporating the quality control metrics previously mentioned will increase accuracy and success rates of manufactured chips.

### Characterization of GFET.

The graphene surface before and after derivatization with PBASE was imaged by atomic force microscopy (Bruker Multimode AFM connected to a Nanoscope V controller imaging in contact mode), and the quality of the graphene surface was characterized by Raman spectroscopy (Renishaw inVia microscope using laser wavelength of 532 nm). Electrical characteristics of the fabricated GFETs were quantified by performing current (*I_DS_*)–voltage (*V_DS_*) sweeps (*V_DS_*: in the range of 0 to 100 mV), at variable gate bias (*V_GS_*, range of 0 to −1 V) in an Agilent 1500B Semiconductor Parameter Analyzer. The gate bias sweeps were done both forward and backward (from 0 to 1 V at 0.2-V increments). The minimum in the *I_DS_* vs*. V_GS_* plot, at a constant *V_DS_*, was recorded as the Dirac voltage (*V_D_*), which provides a measure of the relative charge on the surface and a metric for further biological assays. As a proof of concept of the sensor and probe selection, initial tests were performed on Keithley 2400/2602B source meters at 30 mV *V_DS_* for cyclic measurements. The remaining tests were performed on a custom homemade electronic reader (PIVOT reader) whose construction is outlined below.

### Fabrication of Custom PIVOT-GFET Reader.

As portability and POC testing are one of the main goals of this work, we designed and fabricated a custom miniaturized source-measure device termed a PIVOT reader. The device consists of analog circuitry connected to a microcontroller–Analog to Digital converter (ADC) system that generates a triangular voltage waveform (−0.5 to +1.5 V at the ramp rate of 0.1 V/s) that is fed into the gate terminal of the GFET for the *V_GS_*. The microcontroller–Digital to Analog Converter (DAC) subsystem also provides the *V_DS_* bias voltage. The *I_DS_* was measured continuously through a transimpedance amplifier circuit whose output is read through the ADC–microcontroller subsystem. The device is capable of WiFi connectivity (*SI Appendix*, Fig. S3).

### Aptamer Selection.

Several groups have reported aptamer-based diagnosis and therapeutics (16–18) relevant to SARS-CoV-2. However, those studies have used denatured products, such as RNA, N, and S proteins of the viruses as the analytes. No aptamer-based study is reported for the whole virus of the infective viral species. The current study was designed to use intact whole virus as an analyte for COVID-19 testing. Based on extensive literature review (16–18), we shortlisted six different RNA and DNA aptamers developed for SARS-CoV and SARS-CoV-2 with high affinity (*SI Appendix*, Table S1): two RNA aptamers (18), two DNA aptamers (17) for N protein, and two DNA aptamers for RBD of SARS-CoV-2 (16). The aptamer-S1, aptamer-N1, aptamer-N2, and aptamer-N3 reported in *SI Appendix* showed less affinity to the receptor-binding region S protein and N proteins of SARS-CoV-2. Among the aptamers analyzed in *SI Appendix*, Table S1, the aptamer with highest affinities, Aptamer-S for RBD (*K*_d_ ≈ 5 nM) and Aptamer-N for nucleocapsid protein (*K*_d_ ≈ 0.5 nM), were selected for the final study. (See *SI Appendix*, Fig. S1 for 2D structures of aptamers.) Scrambled variants of individual aptamers were also used to check for their relative response compared to normal S and N aptamers. The scrambling is carried out by pseudorandomization of the respective N- and S-aptamer nucleotide sequences.

### Aptamer Derivatization on GFET.

The amine-derivatized aptamers were dissolved in 1× PBS buffer containing 0.5 mM MgCl_2_ and thermal annealing was performed by controlled heating at 94 °C for 2 min and slow cooling to room temperature. The annealed aptamer was stored at −20 °C for further usage. Early experiments indicated that cations have a significant impact on *V_D_* shift when there has not been adequate ionic incubation post functionalization (*SI Appendix*). A large portion of the experiments were done in 0.1× PBS and 0.9% vol/vol saline to use a lower ionic strength and experiments indicate little difference in effectiveness between saline and PBS. The functionalization was performed by adding ∼10 μL of 1 µM of aptamer on PBASE-functionalized GFET for 60 min. Excess aptamer was washed with 1× PBS (or saline) and unreacted PBASE was passivated using ∼10 μL of 10 mM ethanolamine (EA) solution for 45 min. The excess EA was washed, and chips were incubated in 1× PBS, 0.5 mM MgCl_2_ (or saline) over 24 h to limit ionic drift (*SI Appendix*, Fig. S4). This incubation step was used to stabilize the GFET against ionic drift, which is a shift in Dirac potential caused by the permeation of ions through graphene, countering the graphene p-doping. One criterion used in data verification was less than a 5% change in *V_D_* under PBS incubation over 10 min.

### Antigen Protein Detection Using S- and N-Aptamer–Derivatized GFETs.

A baseline measurement (without adding any antigens) of *I_DS_* vs*. V_GS_* was taken on the aptamer-functionalized GFET by sweeping the *V_GS_* within the range of ±0.5 V while *V_DS_* was maintained at 100 mV. The baseline measurement was done in 0.1× PBS (or saline) and the GFET was allowed to stabilize over a period of ∼30 min. Chips that had greater than a 5% *V_D_* shift from the last 10-min interval were excluded from data analysis as the ionic drift would skew the results. This value was noted as *V_D0_*. To analyze the concentration-dependent sensor response, different concentrations of cognate proteins’ RBD in the range of 10, 20, 50, 100, and 200 nM and N protein in the range of 1, 5, 10, 20, 50, and 100 nM (17) were added to the sample well of the GFET chip. After 10 min incubation, excess unbound protein was washed three times using PBS buffer and an *I_DS_* vs*. V_GS_* measurement was performed again, and the Dirac voltage is noted as *V_D_*. The response, through the *V_D_* shift, was then calculated according to the following relationship:

[1] $$\Delta \;V_{D}=V_{D}-V_{D0}.$$

### Data Analysis/Data Rejection Criteria.

To ensure optimal data consistency, few data rejection criteria were set up; high emphasis was placed on the nature of the *I_DS_* vs. *V_GS_* curves that were collected—any deviation in the shape of the curve from the ideal “V”-shaped curve (characteristic of ambipolar charge carriers in graphene) was considered a bad test and the data were discarded from further consideration. *I_DS_* vs. *V_GS_* curves that showed a high coefficient of variation (>5%) among multiple *V_GS_* sweeps were also discarded.

As an extension, we wanted to check whether the sensor could detect different variants of SARS-CoV-2. To that end, we tested sensor response using SARS-CoV-2 variants such as B.1.1.7 (N501Y), Y453F, D614G (33, 46, 47), and Omicron (B1.1.529) using different concentrations of recombinant RBD (or Spike in the case of D614G and Omicron) proteins. Two different concentrations (100 fM, 100 pM) of mutant variants were used and the sensor response was compared with wild-type cognate RBD of SARS-CoV-2.

### Inactive Virus Detection in Simulated Conditions.

Experiments were performed by preparing diluted solutions containing heat-inactivated SARS-CoV-2 (wild type, alpha variant) USA-WA1/2020**, 9.55 × 10^6^ TCID_50_/mL (Zeptometrix). The concentration indicated in PFU/mL was calculated as described by Ding et al. (48). We have measured the effect of increment of virus dilution (6.68 PFU/mL to 6.68 × 10^6^ PFU/mL) on GFET sensor response. Additional tests were done with Delta variant heat-inactivated virus (2 × 10^9^ copies per milliliter ORF1a). A 10,000× dilution of virus that is a 200 copies per microliter concentration was used. Sample detection took an initial 30-min incubation period with 1× PBS (with 0.5 mM MgCl_2_) for aptamer stabilization. The *V_D_* was measured in 10-min intervals after which the sample was added and incubated for 20 min before the final reading was obtained. The difference in Dirac point between the baseline measurement and the sample at 20 min was used in data analysis.

### GFET Sensor Specificity Analysis.

To analyze the specificity of the GFET sensor, two different concentrations of cognate proteins for MERS-CoV, SARS-CoV, and SARS-CoV-2 were analyzed. Sensor responses at an ultralow concentration (100 fM) as well as in the saturation ranges (100 nM) using aptamers for RBD and N protein were investigated. To better simulate the conditions under which actual viral samples exist—nasopharyngeal swabs and oral saliva swabs—we prepared a “sample buffer” by collecting saliva samples from RT-PCR confirmed negative individuals, diluting it by 10 times in 1× PBS buffer. In the next step, concentration-dependent response analysis was performed by using different dilutions of inactive MERS-CoV and SARS-CoV-2 viruses (670 PFU/mL to 6.7 × 10^5^ PFU/mL) in the sample buffer. These dilutions were then directly added to the GFET sample reservoir and response was recorded.

### On-Field Clinical Sample Testing.

To check the potential of the GFET sensor to discriminate between SARS-COV-2–positive and –negative patient samples, clinical on-field testing was carried out in two phases using samples collected in two different locations—first during the pandemic dominated by the original (wild-type) SARS-CoV-2 virus (Chicago, IL, March 2021) and next during the pandemic period dominated by the Delta/Omicron variants (Las Vegas, NV, 6 to 8 January 2022).

During the original testing phase, the oral swab samples of patients were collected by a trained clinician in 3 mL of (0.9% wt/vol) saline (approved by CDC) and further RT-PCR was performed by CLIA (Clinical Laboratory Improvement Amendments)-certified Optima Laboratory Inc. in Chicago, IL. Upon sample collection each patient sample was given a serial number effectively deidentifying patient information. No patient identifiable information was used during the data analysis. During the recent clinical testing at the peak of the Delta/Omicron variant, samples were collected using oral swabs (saliva samples) in 400 µL 0.1× saline solution and nasal swabs for PCR test validation. After sample collection, 10 µL of the viral sample in saline was directly added to the GFET well and data were recorded through the PIVOT-GFET reader. Clinical sample analyses conducted on the mass-produced chips were done with a 48-h 1× PBS 0.5-mM MgCl_2_ incubation step in the functionalization process.

RT-PCR analysis was performed using the FDA-approved Promega RT-PCR test kit for SARS-CoV-2. Because Aptamer-S showed higher sensor response with inactive virus in a simulated environment, it was used for all of the initial phase of patient sample diagnosis. A total of 30 patient samples (initial phase, Chicago, IL) and 26 patient samples (second phase, Las Vegas, NV) were tested and compared retrospectively with RT-qPCR (Ct value ≤35) data. The known RT-qPCR–negative data were used to set the sensor response threshold value with 99.7% CI using ±3σ analysis to predict negative patient samples. During each phase of the field testing, the sensor response value above the mean +3σ was assigned as positive for the respective phase. The rationale behind the separate analysis of each phase analysis is to reduce the variability in GFET fabrication, sample variability, handling, and virus mutation-mediated affinity for the probe during two different phases of testing. We compared our results with the PCR results, obtained in parallel, by calculating the percent agreement between our test results and those of the PCR. Percent agreement is a method of comparing the results of two diagnostic tests, where positive and negative indicate the percentage of positive/negative results that agree between the two tests. The PPA and NPA of the tests were calculated as described by FDA guidelines (49) and according to Eq. **2**:

[2] $$\mathrm{PPA}=100\times \frac{a}{a+c},\;\mathrm{NPA}=100\times \frac{d}{b+d}.$$

Here, *a* is the positive results indicated by our GFET sensor that coincide with a positive PCR result, *b* indicates the number of positive PIVOT results with a negative PCR result, *c* is the negative results with a positive PCR result, and *d* is the negative results with a negative PCR result. The LoD and LoQ of the sensor were estimated using SD of the response and the slope method (49). All the data presented are the mean of at least three measurements and an error of one SD.

## Supplementary Material

Supplementary File

## Data Availability

All study data are included in this article and/or *SI Appendix*. Raw data/files are available upon request to the corresponding authors.
