# Supplementary material for: Rapid self-test of unprocessed viruses of SARS-CoV-2 and its variants in saliva by portable wireless graphene biosensor
Source: Proc Natl Acad Sci U S A. 2022 Jun 28;119(28):e2206521119. doi: 10.1073/pnas.2206521119 (PMC9282385; doi:10.1073/pnas.2206521119)
Supplement: Supplementary File [file pnas.2206521119.sapp.pdf]

PNAS

Supplementary Information for

Rapid self-test of unprocessed viruses of SARS-CoV-2 and its variants in saliva by portable wireless graphene biosensor

Deependra Kumar Ban[1,a], Tyler Bodily[1,b], Abhijith G Karkisaval[1,a], Yongliang Dong[a], Shreyam Natani[a], Anirudh Ramanathan[b], Armando Ramil[b], Sunil Srivastava[e], Prab Bandaru*[a,c], Gennadi Glinsky*[d], Ratnesh Lal*[a,b,c]

[a]Department of Mechanical and Aerospace Engineering, [b]Department of Bioengineering, [c]Materials Science, [d]Institute of Engineering in Medicine, University of California, San Diego, 92093, USA, [e]Ampera Life, Inc, La Jolla, CA 92037

Prab Bandaru*, Gennadi Glinsky*, Ratnesh Lal*

Email: pbandaru@ucsd.edu, gglinskii@ucsd.edu, rlal@eng.ucsd.edu

This PDF file includes:

Figures S1 to S8
Tables S1 to S2
SI References

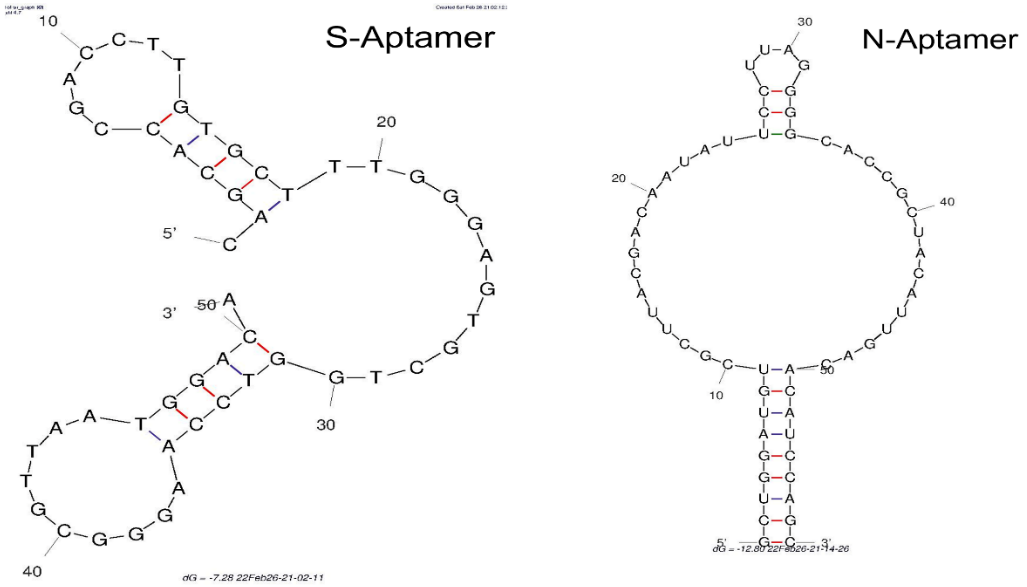

Fig. S1. Equilibrium secondary structures of S-aptamer and N-aptamer obtained from Unafold/mfold [1].

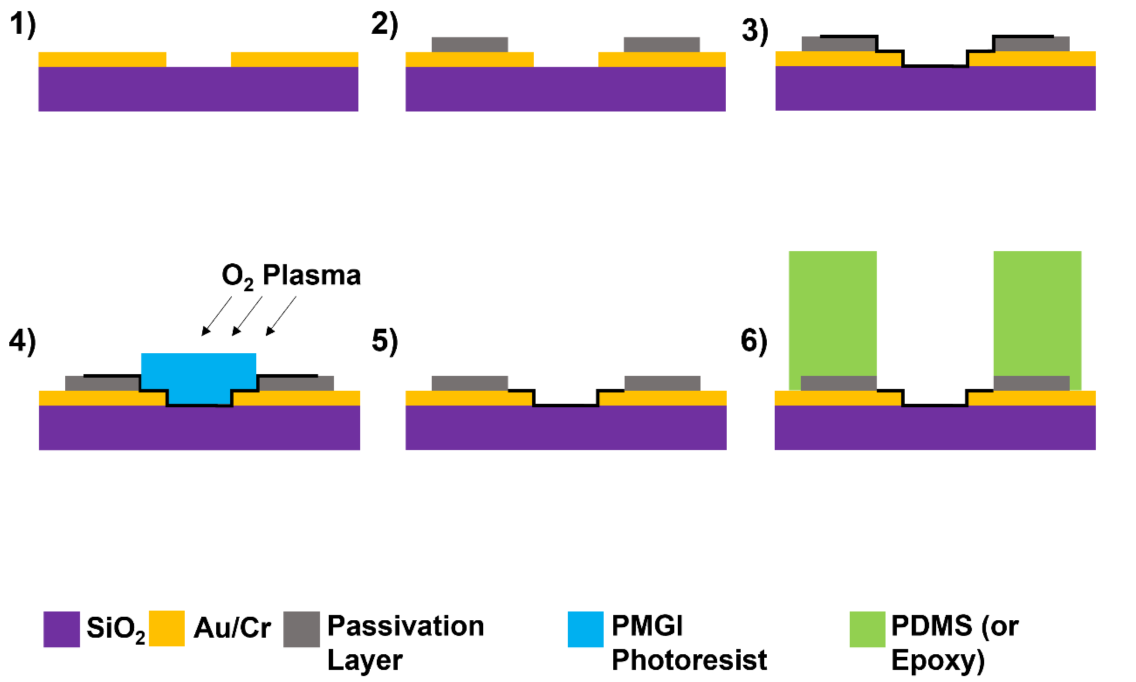

Fig. S2. Schematic representation of graphene Field effect transistor (GFET) fabrication. 1) deposit source drain electrodes (Au/Cr~100 nm) on SiO_2 substrate. 2) deposit passivation layer (SiO_2 or Al_2O_3~80 nm) on source drain electrodes. 3) Wet transfer Graphene onto the patterned substrate, remove PMMA. 4) Apply PMGI photoresist to protect sensor area, remove extra graphene layer by O_2 plasma etching. 5) Lift off the PMGI photoresist and anneal the GFET in forming gas. 6) Apply PDMS or epoxy to form a well for containing sample liquid.

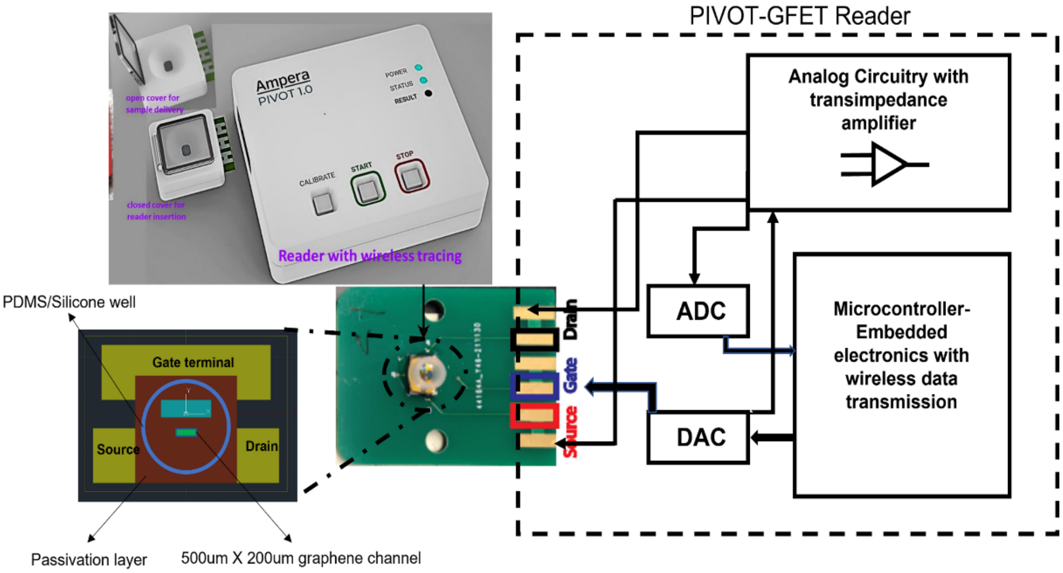

Fig. S3. Device representation, chip schematic and under-the-hood design of the PIVOT-GFET reader. Top left – 3D rendition of the PIVOT-GFET reader device. Bottom left – 2D layout of the microfabricated chip showing source, drain and gate contact pads with PDMS/Silicone well in the center (blue). Bottom center – picture of one of the microfabricated GFET chips mounted on the PCB board that plugs into the device. Right – Internal makeup of the reader device shows the microcontroller subsystem with analog circuitry.

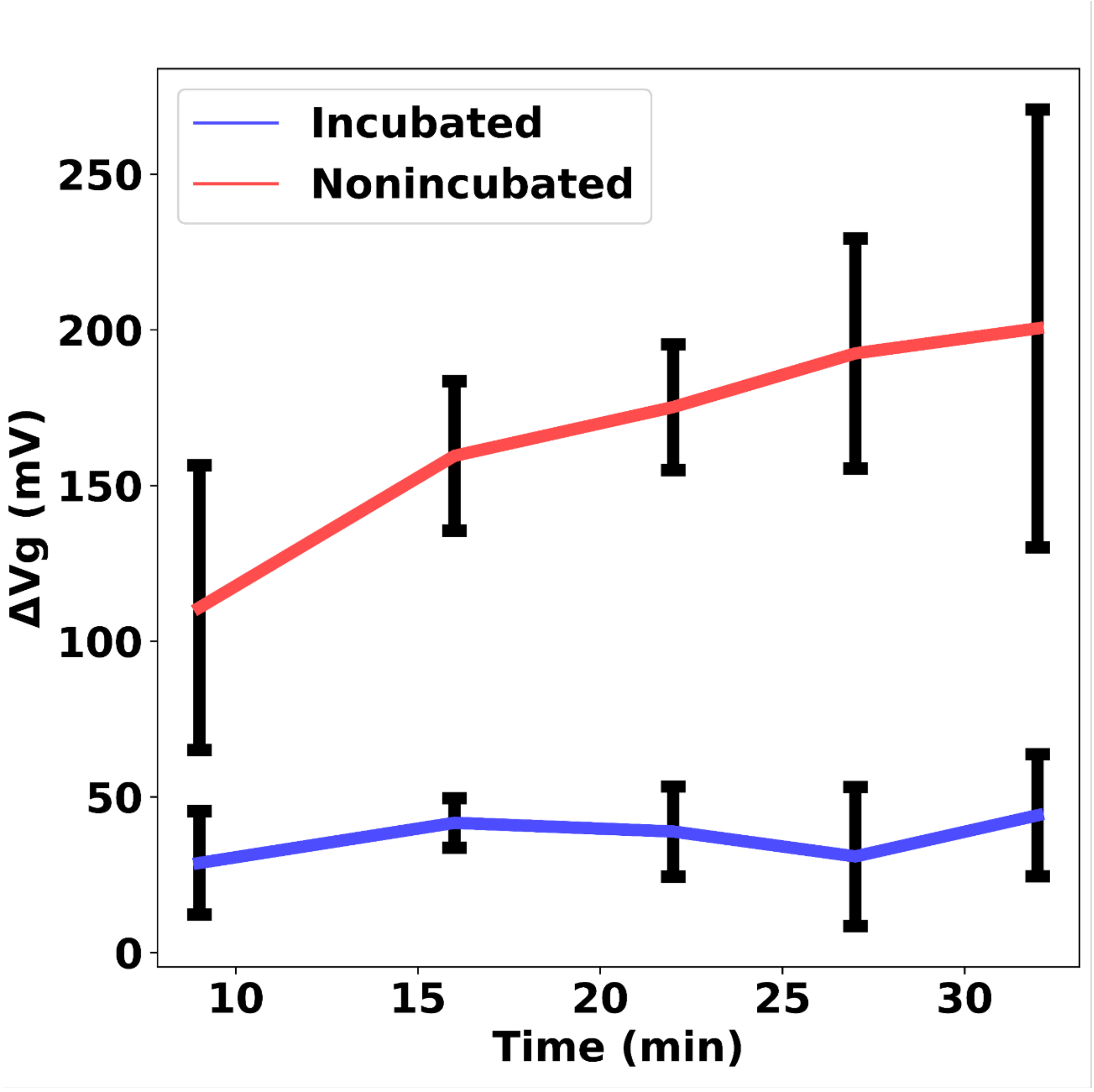

Fig. S4. Ionic drift suppression with 1X PBS incubation overnight.

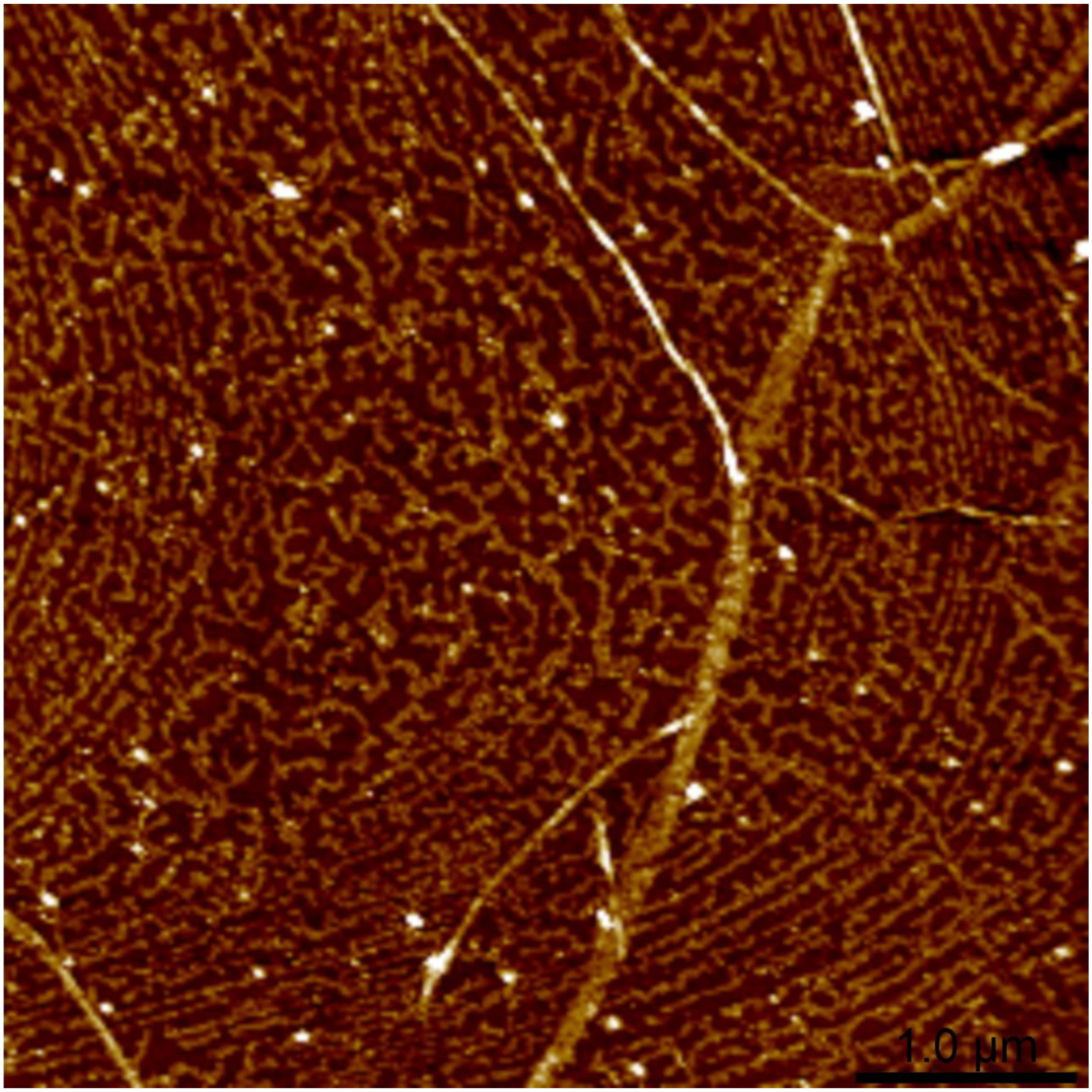

Fig. S5. Low magnification AFM height image of graphene surface showing interconnected grain boundaries. Scale bar denotes 1µm and z-scale = 0-7.5nm

Surface Roughness calculation procedure: After height images were obtained, all images were 1st or 2nd order flattened to remove scan line offsets. The RMS roughness (R_q) is calculated by considering height deviations (Z_i) from the mean image plane data and the whole imaging area (all pixels, N) was considered for the calculation. All roughness calculations were done in Nanoscope Analysis v1.5 software (Bruker Corp.,) The equation used for calculation is as follows:

$$R_q = \sqrt{\frac{\sum z_i^2}{N}}$$

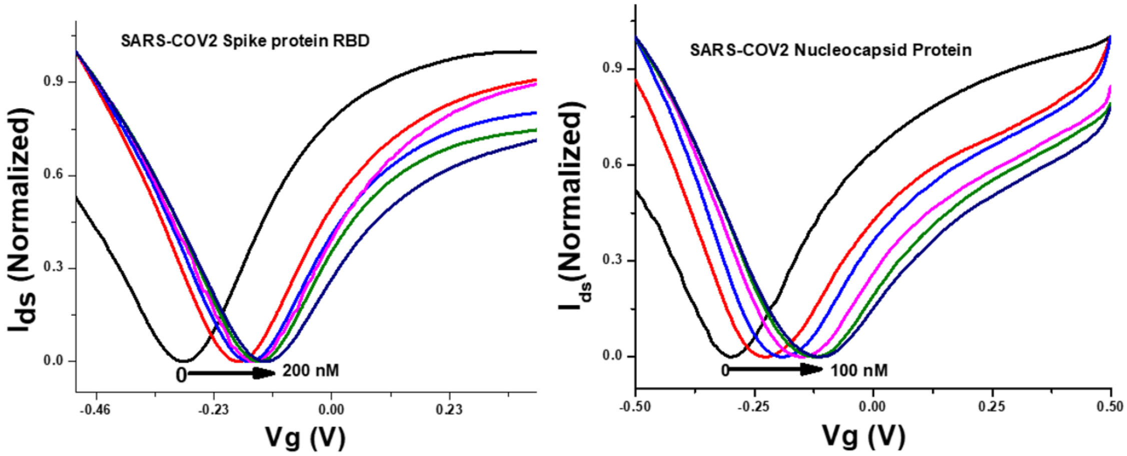

Fig. S6. (A) SARS Cov2 RBD binding mediated transfer curve analysis of Aptamer-S derivatized GFET. (B) SARS Cov2 nucleocapsid protein binding mediated transfer curve analysis of Aptamer-N derivatized GFET.

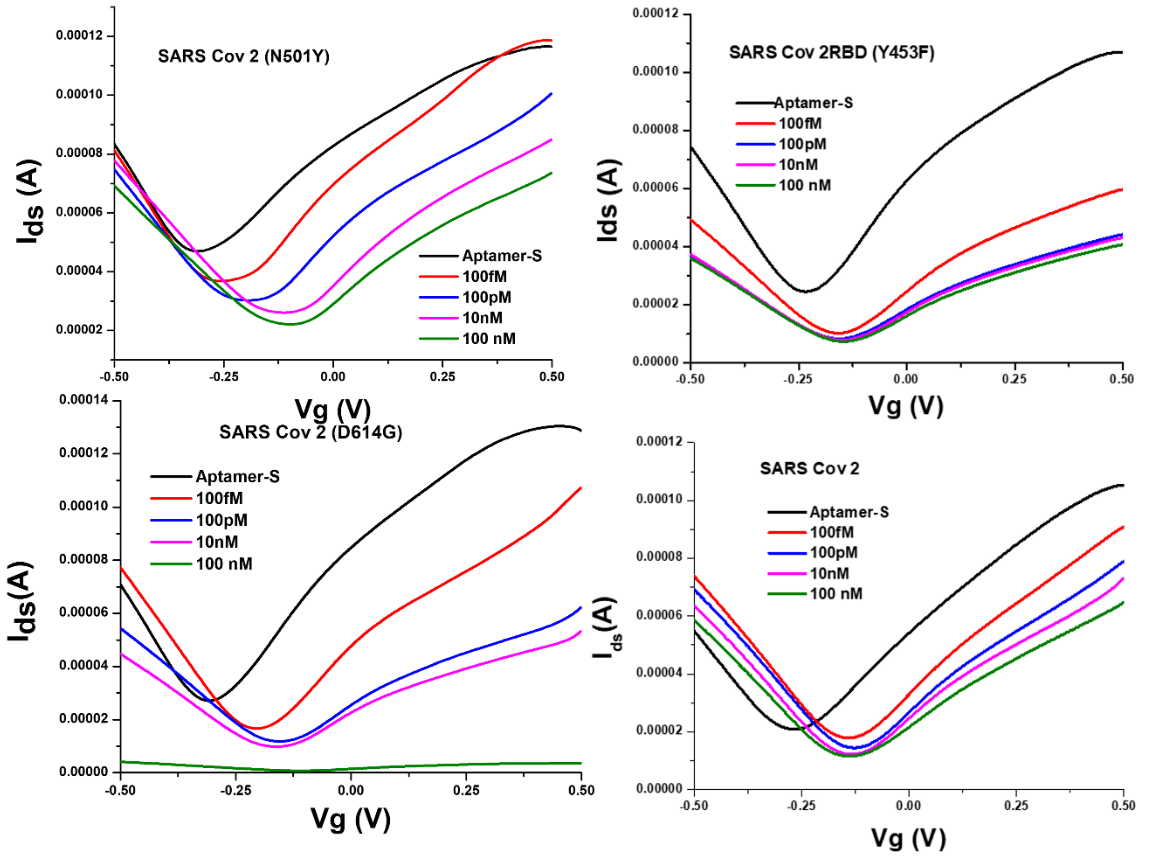

Fig. S7. Detecting the SARS-CoV-2 RBD cognate protein of mutant N501Y, Y453F, D614G, and original virus.

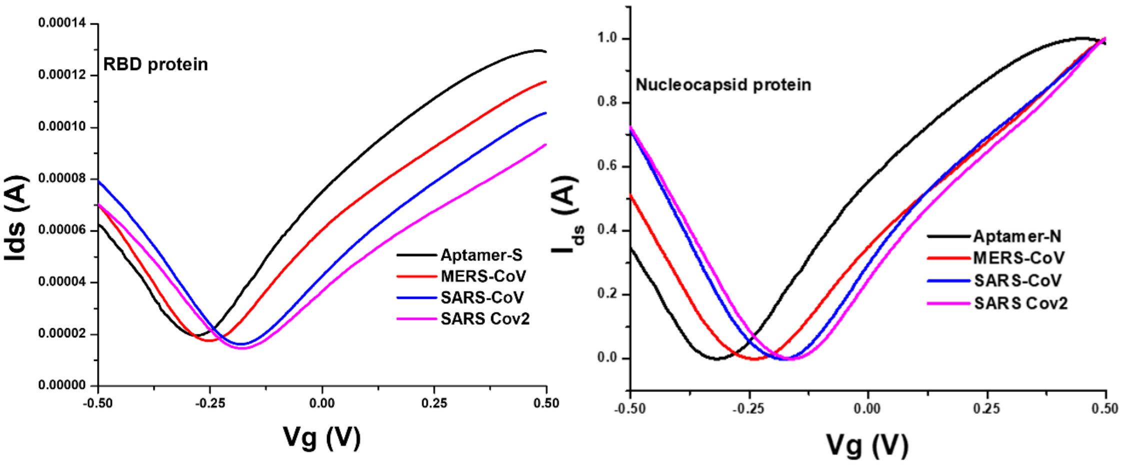

Fig. S8. Analyzing the specificity of the Aptamer-S and Aptamer-N derivatized GFET.

Table S1. Nucleic acid sequences screened for the study

Sequence (5'-3')	Ref.
Aptamer-S: CAGCACCGACCTTGTGCTTTGGGAGTGCTGGTCCAAGGGCGTTAATGGACA	[2]
Aptamer-S1: ATCCAGAGTGACGCAGCATTTCATCGGGTCCAAAAGGGGCTGCTCGGGATTGCGGATATGGACACGT	[2]
Aptamer-N: GCTGGATGTCGCTTACGACAATATTCCTTAGGGGCACCGCTACATTGACACATCCAGC	[3]
Aptamer-N1: GCTGGATGTCACCGGATTGTCGGACATCGGATTGTCTGAGTCATATGACACATCCAGC	[3]
Aptamer-N2: GGGAGAGCGGAAGCGUGCUGGGCCUGUCGUUCGCUGUGUCUUGCUACGUUACGUUACACGGUUGGCAUAACCCAGAGGUCGAUGG	[4]
Aptamer-N3: GGGAGAGCGGAAGCGUGCUGGGCCUCAUUACACACAUCUCACGGGAGACAUAGCUGACGAUAUCCAUAACCCAGAGGUCGAUGG-	[4]
Scrambled Aptamer-S: GGTGGTTTGACTGATCAGAGACTATGCAGCTCTGGCCCGCTAGCCGGAAGT	
Scrambled Aptamer-N: TAGGTATATGTACGCACACCTAAGTCAGCTTGCCCGACGCTGCAGCTCACATGTATGC	

Table S2. Comparison of point of care (POC) antigen test. data collected from the FDA Emergency use Authorization (EUA).

Entity	Target/Readout	LOD	NPA% (n)/PPA% (n)	Time (min)
**PIVOT-Apta-Sensor (current)**	S/N proteins, S Mutant proteins, Droplet, Current-Voltage,	Aptamer-S 1.28 PFU/mL and aptamer-N 1.45 PFU/mL	100 (16)/100 (14)	15<
BinaxNow Ag Card	N protein, N.A., LF,	140.6 $TCID_{50}$/mL	98 (111)/83.3 (50)	15
Ellume	N protein, N.A., LF	$10^{3.80}$ $TCID_{50}$/mL	97 (148)/95 (40)	20
CareStart	N protein, LF, N.A.	8 x 10^2 TCID /mL	99.32 (149)/93.75 (31)	10
Repurpose Glucometer[5]	S/N proteins, aptamer, N.A., Glucometer	6.31 [S]/ 5.27 [N] pM protein	100 (8)/100 (16)	60
Sofia 2	N protein, LF, FL, N.A.	113 $TCID_{50}$/mL	100 (179)/96 (30)	15
LumiraDx	N protein, FLI, N.A.	32 $TCID_{50}$/mL	96.6 (174)/97.6(83)	12

LF= lateral Flow; FL= fluorescent Analysis; FLI= Fluorescence immunoassay, N.A.= Not Applicable

SI References

1. M. Zuker, *Nucleic Acids Research,* **2003,** 31 (13), 3406–3415
2. Y. Song, J. Song, X. Wei, M. Huang, M. Sun, L. Zhu, B. Lin, H. Shen, Z. Zhu, C. Yang, Analytical Chemistry **2020**, 92, 9895.
3. L. Zhang, X. Fang, X. Liu, H. Ou, H. Zhang, J. Wang, Q. Li, H. Cheng, W. Zhang, Z. Luo, Chemical Communications **2020**, 56, 10235.
4. D.-G. Ahn, I.-J. Jeon, J. D. Kim, M.-S. Song, S.-R. Han, S.-W. Lee, H. Jung, J.-W. Oh, Analyst **2009**, 134, 1896.
5. Singh NK, et al., Hitting the diagnostic sweet spot: Point-of-care SARS-COV-2 salivary antigen testing with an off-the-shelf glucometer. *Biosens. Bioelectron.* **38,** 107278 (2021).
